# Priority Control of Intelligent Connected Dedicated Bus Corridor Based on Deep Deterministic Policy Gradient

**DOI:** 10.3390/s25154802

**Published:** 2025-08-04

**Authors:** Chunlin Shang, Fenghua Zhu, Yancai Xu, Guiqing Zhu, Xin Tong

**Affiliations:** 1 College of Transportation, Ludong University, Yantai 264025, China; itsshang@ldu.edu.cn; 2 State Key Laboratory of Multimodal Artificial Intelligence Systems, Institute of Automation, Chinese Academy of Sciences, Beijing 100190, China; yancai.xu@ia.ac.cn; 3 Department of Safety and Emergency Management, Yantai Engineering & Technology College, Yantai 264006, China; zhuguiqing@ytetc.edu.cn; 4 College of Computer Science and Information, Purdue University West Lafayette, West Lafayette, IN 47907, USA; tongxin@alumni.purdue.edu

**Keywords:** urban transportation, bus priority control, deep reinforcement learning, arterial control

## Abstract

To address the substantial disparities in operational characteristics between social vehicles and dedicated bus lanes, as well as the sub-optimal coordination control effects, a comprehensive approach is proposed. This approach integrates social vehicle arterial coordination with bus priority control in dedicated bus lanes. Initially, an analysis of the differences in travel time distribution on both types of roads is conducted. The likelihood of buses passing through upstream and downstream intersections without stopping is also assessed. This analysis aids in determining the correlated traffic states and the corresponding signal adjustment strategies for arterial coordination. Subsequently, an incentive mechanism is established by quantitatively analyzing vehicle delay losses and bus priority benefits based on the signal adjustment strategy. Finally, a deep reinforcement learning framework is proposed to solve, in real-time, the optimal signal adjustment strategy. Simulation experiments indicate that, in comparison to the arterial coordination of social vehicles and dedicated bus arterial coordination control, this method significantly reduces the average per capita delay by 38.63% and 27.43%, respectively, under conventional traffic flow scenarios. This is in contrast to the separate arterial coordination for social vehicles and dedicated bus lanes. Furthermore, it leads to a reduction of 52.17% in the number of bus stops at intersections when compared solely with the arterial coordination of social vehicles. In saturated traffic flow scenarios, this method achieves a reduction in average per capita delay by 29.7% and 9.6%, respectively, while also decreasing the number of bus stops at intersections by 39.5% and 8.7%, respectively.

## 1. Introduction

Dedicated bus lanes represent an effective strategy for mitigating traffic congestion and revolutionizing urban transportation. They have been widely endorsed by governments worldwide. However, the efficiency of bus priority control on arterial lines is still relatively low due to significant differences in signal control between regular vehicle arterial control and bus priority arterial control. To tackle this issue, traditional solutions aim to minimize average delay per person by analyzing delay models, implementing bus pre-signals [1], and incorporating bus priority as one of the index factors for coordinating regular vehicle arterial control. This approach seeks to achieve compatibility between regular vehicle arterial green wave and bus priority. Buses are known for their randomness and fluctuation characteristics, leading to a wide distribution along roads. Consequently, there is often underutilization of signals and poor effects of bus priority, posing significant challenges for traffic management. To address these issues, some scholars have proposed optimizing the intersection characteristics of buses through measures such as speed guidance [2], proactive priority [3], combining speed guidance with signal adjustments [4] based on the coordination of regular vehicle arterial control. However, this approach also brings about challenges, such as high demand for hardware and software equipment in the initial stages and significant impacts on the coordination of regular vehicle arterial control. Furthermore, with the continuous increase in length and density of bus routes, the associated traffic state information has become high-dimensional and continuous, further increasing the difficulty in making real-time and accurate traffic signal decisions.

Reinforcement learning has made significant progress in research on speed control, path decision-making [5], traffic status prediction, and distributed signal optimization [6] due to its real-time decision-making advantages. However, traditional reinforcement learning faces challenges in handling high-dimensional and continuous traffic state information, which limits its real-time decision-making optimization. To address this issue, some scholars have turned to deep reinforcement learning by leveraging the data processing capabilities of deep learning in transportation research. For example, the authors of [7] apply deep reinforcement learning to improve traffic efficiency at intersections through traffic signal decision-making. Additionally, the authors of [8] propose a tram signal priority control strategy based on deep reinforcement learning to address coordination issues between trams and social vehicles.

This paper focuses on analyzing the operational status of arterial sections for social vehicles and buses and proposes an integrated control method for arterial coordination that combines social vehicle arterial coordination with bus priority without disrupting the overall coordination of social vehicle arterials. The multi-step control problem of traffic signals for buses at intersections is transformed into a Markov Decision Process while considering the impact of signal adjustments on upstream and downstream intersection traffic states. The objective is to optimize both the average delay per person and the number of stops along the entire route by developing a dedicated lane bus priority strategy based on iterative analysis using deep reinforcement learning.

In conclusion, this paper presents two primary contributions.

(1) A chain-type green-wave control method is proposed to address the coordination challenges between the social vehicle arterial line and bus arterial line. This method involves alternating between executing the scheme of arterial coordinating demands of social vehicles and dedicated buses, thereby overcoming the limitations of a single arterial coordination scheme.
(2) To tackle the issue of poor real-time decision-making in high continuous states, a deep reinforcement learning chain green-wave control model suitable for high-dimensional continuous traffic conditions is provided. This model enables real-time decision-making with continuous conditions and actions.

The structure of this paper is as follows: Section 2 outlines the research framework, Section 3 introduces the switching method for arterial signal control scheme, and Section 4 analyzes the application of deep reinforcement learning for dedicated bus arterial control, followed by simulation results in Section 5. Finally, fundamental conclusions are presented in Section 6, and potential future research directions are discussed.

## 2. Literature Review

**Figure 1:**
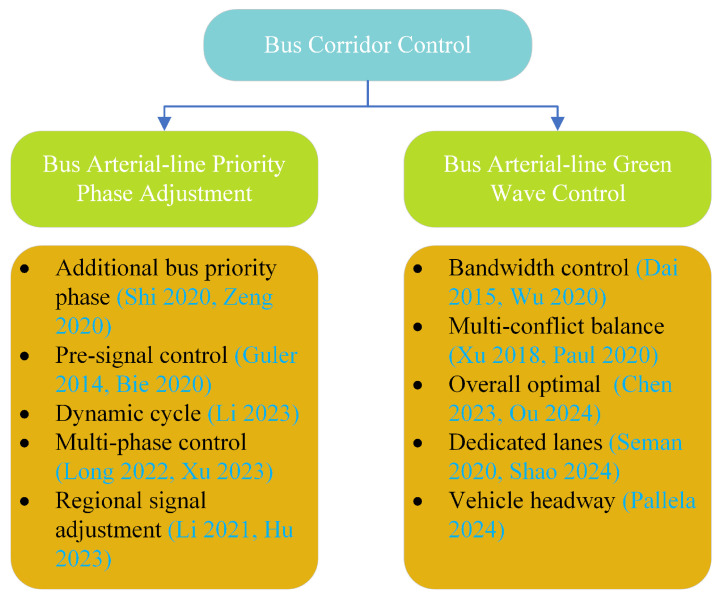
Summary of relevant research. Sources: Shi (2020) [9], Bie (2020) [10], Zeng (2020) [11], Guler (2014) [1], Li (2021) [12], Long (2022) [13], Li (2023) [14], Xu (2023) [15], Hu (2023) [16], Dai (2015) [17], Xu (2018) [18], Wu (2020) [19], Chen (2023) [20], Ou (2024) [21], Shao (2024) [22], Saman (2020) [23], Paul (2020) [24], Pallela (2024) [25].

With the continuous advancement of bus tracking and positioning technology, as well as the development of dedicated bus lane networks, the coordination and control of bus arterial lines has attracted increasing attention from researchers. Furthermore, a series of research results have been achieved in this area. This article primarily categorizes recent research on the coordination and control of dedicated bus corridors into two categories: one based on coordinated green-wave control of bus arterial lines, and the other based on priority phase adjustment of bus arterial lines. Figure 1 provides an overview of the research interests and methodologies of select scholars.

### 2.1. Bus Arterial-Line Priority Phase Adjustment

Considering the disparities in distribution between bus arrivals and social vehicle arrivals, a single signal control scheme often results in issues such as low traffic efficiency and excessive green light loss. In order to enhance the efficiency of dedicated bus services and optimize the utilization of temporal and spatial road resources, scholars have conducted research on dynamic signal phase priority. Truong [2] proposed a dynamic bus phase adjustment method based on the random arrival characteristics of buses, with the aim of minimizing overall delay on the line. Shi [9] put forward an integrated analysis model that takes into account both bus and private car traffic demands, aiming to minimize weighted delay for buses and passenger cars through passive bus-priority signals and lane-marking measures. Bie [10] integrated intersection bus priority phase adjustment with pre-signal control to develop a coordinated phase control method for main lines. Zeng [11] achieved bus priority and reduced average delay per passenger at the route level by granting bus signal priority. Li [12] suggested a regional coordinated bus-priority signal control method based on the arrival time and delay of buses on mainlines with general traffic, resulting in lower average per capita delay. Long [13] introduced an extended dueling double deep Q-learning algorithm for TSP strategy in a connected environment, considering multiple conflicting bus priority requests, as well as constraints of traffic signals and phase-skipping rules to reduce delays for passengers using buses. Li [14] implemented dynamic cycle adjustments through a bi-level programming model based on the dynamic cycle and arrival rate of private vehicles in a connected environment, combining analysis of private vehicle arrivals with signal phase adjustments. Xu [15] proposed a multi-agent-based control method to develop a transit signal priority (TSP) scheme for urban traffic networks. This approach combines transit priority needs with green-light-phase adjustments in order to enhance bus transit efficiency. Hu [16] developed a bus priority control optimization model for conditions without dedicated bus lanes, utilizing the Frank–Wolfe algorithm to optimize carbon emission reductions for buses and various types of social vehicles under different circumstances. These research advancements have significantly improved public transportation efficiency. However, the implementation of these studies necessitates real-time traffic data and places high demands on road hardware configurations, thereby presenting challenges for widespread urban adoption and application.

### 2.2. Bus Arterial-Line Green-Wave Control

Arterial-line green-wave control has traditionally been the primary method for improving the efficiency of arterial-line traffic. However, with advancements in real-time vehicle positioning technology and artificial intelligence algorithms, it is now possible to process dynamic bus information in real time. As a result, several scholars have conducted research on bus arterial-line green-wave control by integrating strategies such as vehicle state monitoring, vehicle state forecasting, and signal control. Dai [17] developed a new green-wave control method for bus corridor coordination. This method analyzes the coordinated and priority demands of social and bus vehicles through comprehensive analysis. Xu [18] created a two-layer planning model focusing on speed adjustment at the upper layer and intersection timing at the lower layer to alleviate conflicts between bus-priority signals and social vehicle corridor coordination signals. Wu [19] proposed a dynamic coding solution framework to optimize dedicated bus lanes while considering the speed difference between buses and social vehicles. This approach aims to optimize bus headway and reduce signal control conflicts between buses and social vehicles. Chen [20] introduced a bus delay minimization index into the coordination of main arteries for general traffic with the goal of improving the efficiency of bus transit on main lines and optimizing bus operation speeds. Ou [21] proposed a Stochastic Priority-Integrated Signal Coordination (SPIC) method based on arterial signal coordination that effectively reduces delays in bus operations. Shao [22] utilized Vehicle-to-Everything (V2X) technology to introduce intermittent exclusive lanes for buses based on dedicated lanes with an aim to enhance road segment capacity by improving dedicated lane usage efficiency.

The prioritized control of bus arterial lines often involves multiple related elements and high decision-making dimensions. Some scholars have studied the coordination of bus arterial lines from the perspective of multi-objective collaborative optimization. Seman [23] proposed a collaborative control model for dedicated bus lane cluster problems, guaranteeing vehicle headway and bus priority, avoiding bus clusters, and optimizing passenger waiting time. Anderson [24] introduced an optimization model based on Brownian motion to enhance the efficiency of conditional signal priority by comprehensively analyzing factors such as schedule settings, bus speed, and bus headways. Thodi [26] demonstrated an analytical approach by deriving closed-form expressions for optimal red truncation and green extension for a two-phase signal using cumulative count curves. This approach addressed real-time computation and solution optimality issues in existing priority systems. Pallela [25] proposed a bus priority model for arterial roads using fundamental diagram theory, integrating factors such as main station time, headways, and arterial flow into the analysis.

### 2.3. Summary

In conclusion, scholars worldwide have conducted extensive research on the coordination of bus arterial lines and have achieved significant outcomes. However, there are still some shortcomings that need to be addressed. Existing research methods mainly reactively respond to the operational characteristics of buses. Nevertheless, due to the random and fluctuating nature of buses, their distribution on road sections varies significantly, leading to issues such as low signal-utilization rate and inadequate bus priority effect. Real-time optimization of signal adjustments is challenging using existing research methods. As the length and density of bus routes increase, traffic state information gradually becomes high-dimensional and continuous, further increasing the difficulty of real-time and precise decision-making for traffic signals.

## 3. Outline of Research Framework

### 3.1. Research Framework

The current research on bus priority primarily focuses on implementing strategies such as speed guidance and active signal control. These strategies involve tracking the status of public transportation, providing speed guidance for buses, and coordinating intersection signals to minimize per capita delay. However, these methods often neglect the impact of bus priority on the operation of other vehicles and fail to address the coordination requirements for arterial lines. Furthermore, they heavily depend on real-time monitoring of vehicle and road traffic status, making it challenging to apply them to actual road conditions without expensive hardware equipment and software systems.

This article presents a timely switching control method for the two arterial coordination schemes in conventional bus lanes. It commences by analyzing the operational characteristics of social vehicles and dedicated buses to determine the phase difference between the two arterial coordinations. The state space is then constructed based on the road section bus state and phase differences, and the association constraint relationship of the four actions is determined to support the construction of the action space. Subsequently, this article comprehensively analyzes the per capita delay of the route and the average number of stops at intersections and establishes a reward and punishment mechanism to identify the best switching scheme. Finally, efficient on-demand alternate control of bus arterial coordination and social vehicle arterial coordination schemes is achieved using a deep reinforcement learning framework. This approach not only enhances bus operation efficiency but also optimizes overall line efficiency. The proposed method focuses specifically on conventional bus lanes.

This article focuses on priority control measures for conventional fixed-route, scheduled road buses—comprising diesel, natural gas, and battery-electric buses—that operate within dedicated bus lanes along signalized arterial corridors. Other forms of public transportation, such as metro systems, trams, and trolleybuses, are excluded from the purview of this paper due to their distinct operational characteristics and infrastructure requirements.

### 3.2. Assumptions

This paper makes several assumptions to clarify the scope of the proposed control algorithm.

(1) The focus of this study is a segment of a local arterial road within the bus route network, which is fully equipped with dedicated bus lanes. This design aims to minimize interference from non-public vehicles during bus operations.
(2) The presence of multi-modal vehicle detectors at the entrance and exit of all road sections is assumed in order to obtain the cumulative number of passing vehicles.

The purpose of assumption (1) is to prevent non-public vehicles from interfering with the proposed control algorithm. Assumption (2) is made to specify the necessary hardware requirements for the road environment.

Terminology Clarification:

Buses/Dedicated Buses: This term specifically refers to conventional fixed-route road buses that operate exclusively on dedicated bus lanes, which serve as the primary mode of public transportation in this study.

Social Vehicles: This category includes all non-public transport vehicles, such as private cars, taxis, ride-hailing services, and trucks, that utilize general-purpose lanes.

## 4. Analysis of Operating Characteristics for Dedicated Bus Arterials

This section examines the operational characteristics of bus and social vehicles in terms of their arrival and travel time distributions. It aims to identify the differences between them and utilizes the travel time distribution to determine the timeframe for vehicle arrivals at downstream intersections. This information is crucial for estimating the likelihood of buses passing through downstream intersections without stopping. In light of the constraints posed by adjusting the maximum and minimum green times during signal timing, it is essential to analyze the adjustment constraints for different signal adjustment actions. Therefore, this section primarily delves into analyzing the characteristics of dedicated bus lane intersections and the signal adjustment constraints from two perspectives in order to analyze the features of bus arterial routes.

### 4.1. Analysis of Intersection State Characteristics for Dedicated Buses

**Figure 2:**
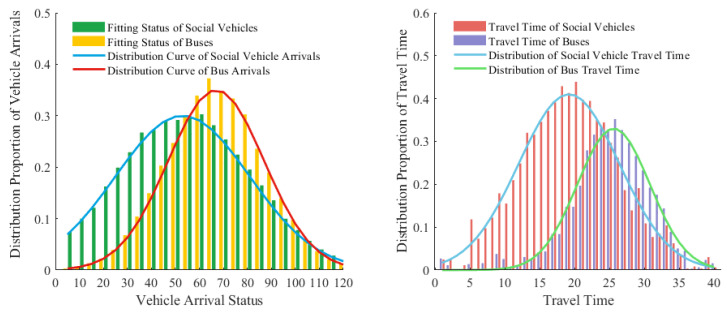
Vehicle characteristic distribution.

In comparison to the travel behavior of social vehicles, the stationary nature of public transportation at stops can lead to disparities in travel time between the two. In order to further investigate these variances, this chapter conducts an analysis utilizing video analysis equipment to examine vehicle arrival and travel time distribution at intersections. Upon conducting a comparative analysis of the travel times of public transportation and buses on road sections (as depicted in Figure 2, data collected in 2022 from Huan Cheng South Road, Kunming, Yunnan), it was observed that bus travel times are delayed in comparison to social vehicles.

A K-S test (shown in the Table 1) was conducted on the travel time of buses, and its asymptotic significance level was found to be 0.101>0.05, indicating that it follows a normal distribution. Therefore, the confidence interval for a confidence level of 1−α can be obtained as follows:

(1) Tt=Tt1,Tt2=n−1S2χα22n−1,n−1S2χ1−α22n−1S2=1n∑j=1n(Xj−X¯)2

where $T_{t1},T_{t2}$ are the upper and lower limits of the confidence interval, $X_{j}$ is the *j*-th sample value, $\bar{X}$ is the sample mean, and *n* is the sample size.

**Figure 3:**
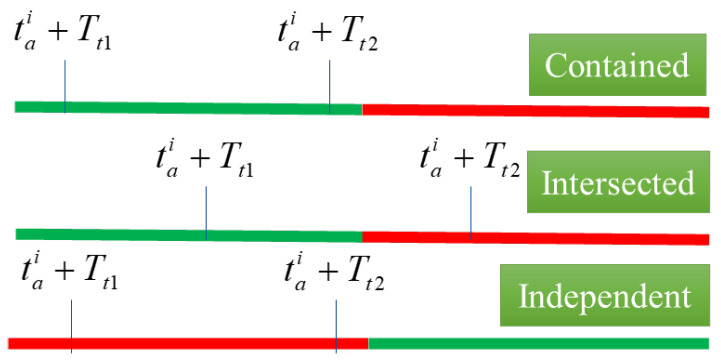
Relationship indication.

Therefore, the arrival time of the bus at intersection i+1 is $t_{a}^{i+1}=t_{a}^{i}+T_{t}$ and $t_{a}^{i+1}\in \left[t_{a}^{i}+T_{t1},t_{a}^{i}+T_{t2}\right]$, while at this time the start and end times of the green light at intersection i+1 are $g_{start}^{i+1}=g_{start}^{i}+\beta_{i}$ and $g_{end}^{i+1}=g_{start}^{i+1}+\Delta g_{i+1}$, with a phase difference of $\beta_{i}$ between road segments, and $\Delta g_{i+1}$ is the duration of the green light at intersection i+1. The time period when the vehicle arrives overlaps with the green light period of the intersection, which can be divided into three cases: contained, intersected, and independent (as shown in Figure 3), and the probability of the bus not stopping at the intersection varies for each case.

(2) $$\lambda_{i}=f\left(\beta_{i}\right)=\left\{\begin{array}{c} \;1,T_{i}\in \left[g_{start}^{i+1},g_{end}^{i+1}\right]\\ \;0,T_{i}\notin \left[g_{start}^{i+1},g_{end}^{i+1}\right]\\ \frac{g_{end}^{i}-t_{a}^{i}-T_{t1}}{T_{t2}-T_{t1}},others \end{array}\right.$$

Bus stop dwellings (including factors such as stop types and passenger boarding and alighting) are primarily responsible for variations in travel time distribution. This model quantifies their stochastic impact on green wave coordination through the confidence interval $T_{i}$.

The confidence interval $T_{i}$ is derived from measured data and reflects operational characteristics under prevailing environmental conditions. In instances of substantial external environmental changes (e.g., snowfall, freezing rain, and other inclement weather conditions), it is essential to update the corresponding distribution parameters alongside new field data to ensure accuracy in the model’s predictions.

### 4.2. Adjustment Strategy for Arterial Signal Control

Through the above introduction, we can obtain the travel time distribution of buses on each road segment, and based on the selection of confidence intervals, we can determine the time range when buses arrive at the road segment. Combining Equation (2), we can also determine the probability of buses passing through downstream intersections without stopping. If there are buses on the road segment, we define Δq>0; otherwise, if there are no buses on the road segment, there are three possible scenarios: no buses on the road segment in reality $\left(\Delta q_{i}=0,\Delta {q_{i}}^{\prime }=0\right)$, one-way bus traffic $\left(\Delta q_{i}\Delta {q_{i}}^{\prime }=0,\Delta q_{i}+\Delta {q_{i}}^{\prime }\neq 0\right)$, or two-way bus traffic $\left(\Delta q_{i}\Delta {q_{i}}^{\prime }\neq 0\right)$. Only the latter two scenarios involve adjustments to signal control plans. We define the early green light start period as $y_{1}=t_{a}^{i}+T_{t1}-g_{start}^{i+1}$ and the green light extension period as $y_{2}=g_{end}^{i+1}-t_{a}^{i}-T_{t2}$.

When $\Delta q_{i}\Delta {q_{i}}^{\prime }=0$, it means that at most one direction of road segment a has bus priority demand. Otherwise, both directions of road segment a have bus priority demands. Therefore, combining the constraints of maximum and minimum green signals, the signal adjustment constraints are determined as follows:

(1) When there is a demand for one-way bus priority, that is $\Delta q_{i}\Delta {q_{i}}^{\prime }=0,\Delta q_{i}+\Delta {q_{i}}^{\prime }\neq 0$, it can be mainly divided into the following three situations.

A. If $\lambda_{i}=1$, it means that buses can pass through the intersection during the green light period, so signal adjustment is not required. In this case, the amount of early termination of the red light and the extension of the green light are $\Delta t_{zao}^{i}=\Delta t_{yan}^{i}=0$.

**Figure 4:**
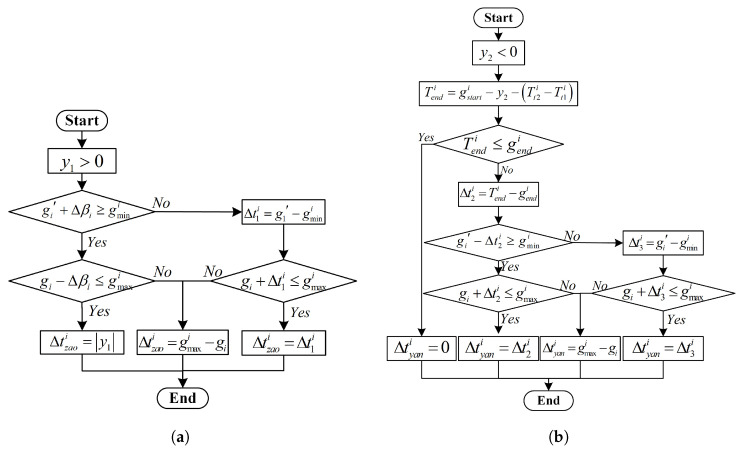
Signal Control Parameter Evaluation Process 1. (**a**) Red light early break analysis. (**b**) Analysis of green light extension.

B. If $\lambda_{i}\neq 1$ and $y_{1}<0$, it means that the green light at the intersection needs to be activated earlier, which requires early termination of the red light. At this time, the green light in the coordinated direction will be lengthened without reducing the green wave bandwidth for other vehicles, thus meeting the requirement of not affecting the coordination of the main road for other vehicles. The control constraints analysis is shown in Figure 4a, where $g_{i}$ is the green light time for this phase, and ${g_{i}}^{\prime }$ is the duration of the adjacent phase’s green light.

C. If $\lambda_{i}\neq 1$ and $y_{2}<0$, the green light at the intersection does not need to be activated earlier, but in order to ensure the travel time for dedicated bus lanes, it is necessary to analyze and judge whether to extend the green light duration for the remaining time. The control constraints analysis is shown in Figure 4b.

(2) When there is a demand for two-way bus priority, that is, $\Delta q_{i}\Delta {q_{i}}^{\prime }\neq 0$, it means that at least one direction has a demand for bus priority. Based on the probability judgment criteria, $\Delta x=\Delta q_{i}\Delta {q_{i}}^{\prime }\left(1-\lambda_{i}\right)\left(1-{\lambda_{i}}^{\prime }\right)$ is determined.

A. If Δx=0, it means that at least one direction does not require signal adjustment, so it is similar to the one-way chain green wave.

B. If Δx≠0, it means that both directions have a demand for signal adjustment, so their demand types are analyzed as follows:

(a) If both directions require early termination of the red light, that is, $\Delta t_{1zao}^{i}\Delta t_{2zao}^{i}\neq 0$, $\Delta t_{1yan}^{i}\Delta t_{2yan}^{i}=0$, then $Max\left(\Delta t_{1zao}^{i},\Delta t_{2zao}^{i}\right)$ is selected as the final adjustment amount;

(b) If both directions require extension of the green light, that is, $\Delta t_{1zao}^{i}\Delta t_{2zao}^{i}=0$, $\Delta t_{1yan}^{i}\Delta t_{2yan}^{i}\neq 0$, then $Max\left(\Delta t_{1yan}^{i},\Delta t_{2yan}^{i}\right)$ is selected as the final adjustment amount;

(c) If one direction requires early termination of the red light while the other direction requires extension of the green light, it is necessary to analyze the relationship between the arrival time $t_{a}^{i},{t_{a}^{i}}^{\prime }$ of the two-way buses at the intersection.

**Figure 5:**
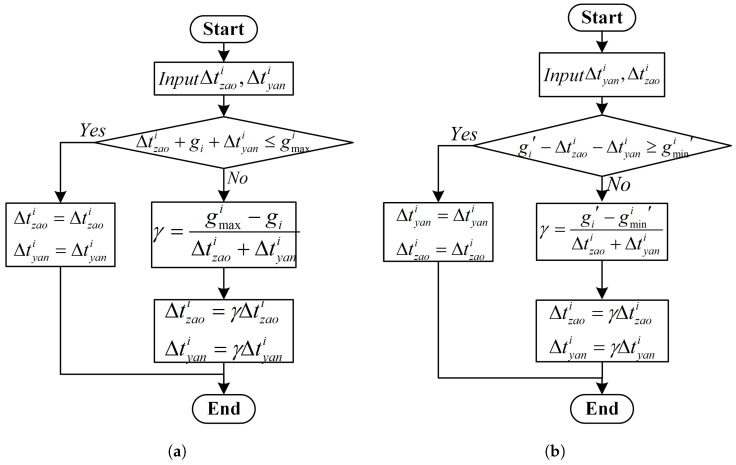
Signal Control Parameter Evaluation Process 2. (**a**) Adjustment during the same green light period. (**b**) Adjustment during the adjacent green light period.

When $0\leq \left|t_{a}^{i}-{t_{a}^{i}}^{\prime }\right|\leq C$, it means that vehicles arrive at the intersection during the same green light period. The judgment rule is shown in Figure 5a, where γ is the proportion coefficient.

When ${g_{i}}^{\prime }\leq \left|t_{a}^{i}-{t_{a}^{i}}^{\prime }\right|\leq C+{g_{i}}^{\prime }$, it means that the vehicles pass through during non-adjacent green light periods and do not interfere with each other, so it is consistent with the constraints of one-way chain green wave.

When ${g_{i}}^{\prime }\leq \left|t_{a}^{i}-{t_{a}^{i}}^{\prime }\right|\leq C+{g_{i}}^{\prime }$, it means that the vehicles pass through during adjacent green light periods. The judgment rule is shown in Figure 5b.

This signal-adjustment strategy is based on relative-time parameters (such as offset $\beta_{i}$ and green-light duration $g_{i}$). Consequently, it is applicable to scenarios involving signal coordination where intersection cycle lengths may differ.

## 5. Arterial Signal Control Method

**Figure 6:**
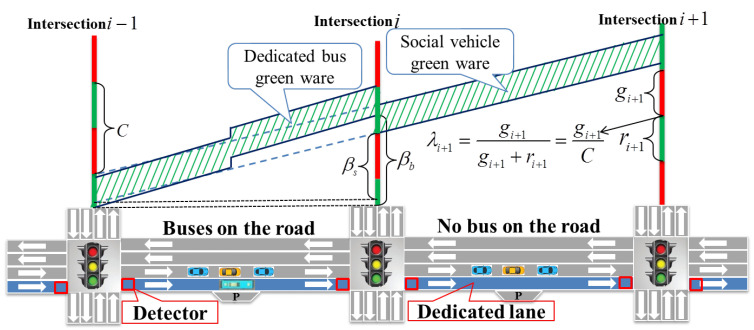
Adjustment during the adjacent green light period.

The key to coordinating and controlling the arterial line is to adjust the signal control strategy of each road section in real time according to the distribution of bus routes. During this process (as shown in Figure 6), it is necessary to assess the execution status of the signals at the road sections and intersections where the buses are located. Therefore, real-time information on bus status can be obtained through onboard GPS data. However, dynamic traffic perception and signal control have high real-time requirements. With changes in the number of arterial sections and buses, signal decision-making becomes a high-dimensional state with multiple states and multiple actions. Therefore, this chapter utilizes deep deterministic policy gradient (DDPG) to address continuous state-space and continuous action-space issues. Next, this chapter will break down the coordination and control problem of the mainline and introduce the state vector, action space, and reward function based on improving bus travel efficiency while minimizing negative impacts on social vehicles.

### 5.1. Stats Space

By comparing the GPS information of buses with the GPS area of the route, the section to which the bus belongs can be determined. As described in Section 4.1, if there is a bus in section *i*, the status of this section is defined as $\Delta q_{i}>0$. By combining with signal control timing at intersections, we can determine the probability of buses passing downstream intersections with Formula (2). If $\lambda_{i}=1$, it means that buses will pass through the intersection without stopping under the existing signal timing, otherwise, it indicates that the buses have a priority demand for traffic signals. Therefore, the real-time status of buses can be grasped by the states of parameters Δq,λ, and the driving status of dedicated bus lanes can be defined as $s=\left(\Delta q,\lambda \right)$, where Δq,λ, respectively, indicate whether there are buses on the road section and the probability of passing through the intersection. The state space of this chapter’s model is defined as $S=\left(s_{1},\ldots ,s_{i},\ldots ,s_{n}\right)$, where *n* represents the number of main road sections.

### 5.2. Action Space

Signal control at intersections needs to be adjusted in real time based on the status of buses, so it is necessary to build a corresponding signal adjustment action correlation model for traffic conditions. As analyzed in section state space, the state of parameter Δq determines whether there is a priority demand for buses, and the state of parameter λ determines the degree of signal adjustment needed. We define the decision action space for the entire main road as $A=\left(a^{1},\ldots ,a^{i},\ldots ,a^{n}\right)$, and $a^{i}$ have four possible signal decision actions for road section *i*, namely $a_{1}^{i}=\left(\Delta t_{zao}^{i},0\right),a_{2}^{i}=\left(0,0\right),a_{3}^{i}=\left(0,\Delta t_{yan}^{i}\right)$, $a_{4}^{i}=\left(\Delta t_{zao}^{i},\Delta t_{yan}^{i}\right)$, where $\Delta t_{zao}$ represents the amount of early termination adjustment of the signal, and $\Delta t_{yan}$ represents the amount of extension adjustment of the signal.

Considering that in the actual operation process, there will be maximum and minimum green light restrictions at each intersection, the signal adjustment has different constraints under different states. Therefore, we define $a^{i}=\mu \left(s_{i}\right)$ to represent the relationship between the action space *A* and the state space *S*, taking into account the specific analysis as follows.

When $\Delta q_{i}\Delta {q_{i}}^{\prime }=0$, only the one-way traffic demand is considered. The relationship between the action space $a_{sole}^{i}$ and the state space in this case is

(3) $$a_{sole}^{i}=\left\{\begin{array}{c} \;a_{2}^{i},\Delta q_{i}\left(1-\lambda_{i}\right)=0\\ \;a_{1}^{i},\Delta q_{i}\left(1-\lambda_{i}\right)\neq 0\&t_{a}^{i}+T_{t1}<g_{start}^{i+1}\\ a_{3}^{i},\Delta q_{i}\left(1-\lambda_{i}\right)\neq 0\&t_{a}^{i}+T_{t2}>g_{end}^{i+1} \end{array}\right.$$

When $\Delta q_{i}\Delta {q_{i}}^{\prime }\neq 0$, the bidirectional traffic demand needs to be considered. The relationship between the action space $a_{double}^{i}$ and the state space in this case is

(4) $$a_{double}^{i}=\left\{\begin{array}{c} \;a_{sole}^{i},\Delta x=0\\ \;a_{4}^{i},\Delta x\neq 0\&y_{1}<0,{y_{2}}^{\prime }<0or{y_{1}}^{\prime }<0,y_{2}<0\\ \;a_{1}^{i},\Delta x\neq 0\&y_{1}<0\&{y_{1}}^{\prime }<0\\ a_{3}^{i},\Delta x\neq 0\&y_{2}<0\&{y_{2}}^{\prime }<0 \end{array}\right.$$

$\Delta x=\Delta q_{i}\Delta {q_{i}}^{\prime }\left(1-\lambda_{i}\right)\left(1-{\lambda_{i}}^{\prime }\right)$, $y_{1}=t_{a}^{i}+T_{t1}-g_{start}^{i+1}$, $y_{2}=g_{end}^{i+1}-t_{a}^{i}-T_{t2}$; $\Delta q^{\prime },\lambda^{\prime }$ represent the parameters of opposite states. It should be noted that the specific value range of the action space $\Delta t_{zao}^{i},\Delta t_{yan}^{i}$ needs to be determined based on the analysis of the maximum and minimum green times for signal control at intersections. The specific judgment method can refer to Section 4.2, which will not be elaborated here.

### 5.3. Reward Function

Public transport signal priority can optimize factors such as bus parking and delay to a certain extent. However, the corresponding increase in public transport priority may have a negative impact on the traffic flow of non-public transport vehicles. Therefore, in order to achieve the best optimization effect, this chapter selects indicators such as per capita delay and bus parking times to construct the action reward function. Signal adjustments will affect the traffic flow of vehicles on road sections. To quantify the impact of signal adjustments on the delay of non-public transport vehicles, this chapter uses the following delay formula:  

(5) $$\left\{\begin{array}{c} \lambda^{i}=\frac{g_{i}}{C}\\ \lambda_{y}^{i}=\frac{g_{i}+t_{zao}^{i}+t_{yan}^{i}}{C}\\ Cost\left(\Delta t_{i}\right)=\bar{d}\left(\lambda_{y}^{i}\right)-\bar{d}\left(\lambda^{i}\right)\\ \bar{d}\left(\lambda \right)=\left\{\begin{array}{cc} \frac{C{\left(1-\lambda \right)}^{2}}{2(1-\lambda x)}+\frac{x^{2}}{2q(1-x)}-0.65{\left(\frac{C}{q^{2}}\right)}^{1/3}x^{2+5\lambda } & \mathrm{if}\;x<0.85\\ \frac{C{\left(1-\lambda \right)}^{2}}{2\left[1-\min(1,x)\lambda \right]}+\frac{Q_{\mathrm{initial}}\cdot T_{a}}{q} & \mathrm{if}\;x\geq 0.85 \end{array}\right. \end{array}\right.$$

$\lambda^{i}$ and $\lambda_{y}^{i}$ denote green ratios before/after adjustment, $Cost(\Delta t_{i})$ quantifies delay change per non-public transport vehicle, *q* is vehicle arrival flow rate (veh/h), *x* is saturation degree (volume-to-capacity ratio), $\bar{d}\left(\lambda \right)$ calculates average delay per vehicle (s) using Webster’s formula for undersaturated conditions (x<0.85) or HCM method for saturated conditions (x≥0.85), $Q_{\mathrm{initial}}$ is initial queue length (veh), and $T_{a}$ is analysis period duration (h). The critical transition at x=0.85 balances model accuracy between operational regimes.

Considering that signal adjustment can improve the traffic efficiency of buses, this paper selects the changes in the traffic state at bus intersections to analyze the impact of signal adjustment. The arrival time of buses before signal adjustment and the green light period at the intersection are $\left[t_{a}^{i}+T_{t1},t_{a}^{i}+T_{t2}\right]$ and $\left[g_{start}^{i},g_{end}^{i}\right]$, respectively. Therefore, the time range for buses to pass downstream intersections without stopping ($t_{wait}^{i-1}=0$) is $\left[c_{i}^{1},c_{i}^{2}\right]=\left[g_{start}^{i},g_{end}^{i}\right]\cap \left[t_{a}^{i}+T_{t1},t_{a}^{i}+T_{t2}\right]$, and the time range for buses to pass downstream intersections without stopping after signal adjustment is $\left[c_{iy}^{1},c_{iy}^{2}\right]=\left[g_{start}^{i}+t_{zao}^{i},g_{end}^{i}+t_{yan}^{i}\right]\cap \left[t_{a}^{i}+T_{t1},t_{a}^{i}+T_{t2}\right]$. When buses stop and wait at upstream intersections ($t_{wait}^{i-1}\neq 0$), the time range for buses to pass downstream intersections without stopping after signal adjustment is $\left[c_{iy}^{1t},c_{iy}^{2t}\right]=\left[g_{start}^{i}+t_{zao}^{i},g_{end}^{i}+t_{yan}^{i}\right]\cap \left[t_{a}^{i}+t_{wait}^{i-1}+T_{t1},t_{a}^{i}+t_{wait}^{i-1}+T_{t2}\right]$. Based on these, the state transition matrix after signal adjustment can be obtained, and it is found that the probability distribution of the next state can only be determined by the current state, and all previous states in the time series are irrelevant.

(6) $$P_{i}=\left[\begin{array}{cc} \;\eta_{tt}^{i} & \eta_{nt}^{i}\\ \eta_{tn}^{i} & \eta_{nn}^{i} \end{array}\right]=\left[\begin{array}{cc} \;\frac{c_{iy}^{2}-c_{iy}^{1}}{T_{t2}-T_{t1}} & \frac{c_{iy}^{2t}-c_{iy}^{1t}}{T_{t2}-T_{t1}}\\ 1-\frac{c_{iy}^{2}-c_{iy}^{1}}{T_{t2}-T_{t1}} & 1-\frac{c_{iy}^{2t}-c_{iy}^{1t}}{T_{t2}-T_{t1}} \end{array}\right]$$

$\eta_{tt}^{i}$ represents the probability of uninterrupted passage for both upstream and downstream. $\eta_{tn}^{i}$ represents the probability of uninterrupted passage upstream while waiting for downstream to stop. $\eta_{nt}^{i}$ represents the probability of stopping upstream while waiting for downstream to pass through without stopping, and $\eta_{nn}^{i}$ represents the probability of both upstream and downstream stopping and waiting.

From the above analysis, it is evident that the probability distribution of transitioning to the next state from the current state can only be determined by the current state, with previous states in the time series being irrelevant. Therefore, it exhibits the Markov property and can be analyzed using a Markov chain. As buses do not stop at intersections, there will be cumulative optimization in terms of travel time. The impact of signal adjustment on buses can be defined as

(7) $$\left\{\begin{array}{c} Gain\left(\Delta t_{i}\right)=\Delta \eta_{tt}^{i}\left(\bar{t_{wait}^{i-1}}+\bar{t_{wait}^{i}}\right)+\Delta \eta_{tn}^{i}\bar{t_{wait}^{i-1}}+\Delta \eta_{nt}^{i}\bar{t_{wait}^{i}}\\ \Delta \eta^{i}=\left(1-\alpha \right)\left[P_{i}-P_{i}\left(\Delta t_{i}=0\right)\right] \end{array}\right.$$

$\bar{t_{wait}^{i}}$ is the average delay time of buses at the intersection, which can be calculated based on historical statistical data; $\Delta \eta^{i}$ represents the probability change before and after signal adjustment.

According to Formulas (5) and (7), the change in average delay per person on the bus route can be obtained as

(8) $$Y_{y}=\sum_{i=1}^{N}\frac{Mq_{bus}^{i}Gain\left(\Delta t_{i}\right)-Cost\left(\Delta t_{i}\right)q_{i}}{q_{i}+M}$$

$q_{bus}^{i}$ is the carrying capacity of bus passengers, which can be obtained through data from card swiping records of passengers getting on and off the bus; $q_{i}$ represents the traffic flow of social vehicles.

Through Formula (6), we can obtain the probability of buses passing through intersections without stopping. Therefore, for the entire bus route, the change in the number of times buses stop at intersections can be obtained as

(9) $$Y_{s}=\sum_{i=1}^{N}\Delta \eta^{i}$$

Finally, based on Formulas (8) and (9), and combined with normalization analysis, the reward function is obtained as

(10) $$\left\{\begin{array}{c} r=\lambda_{1}\delta_{1}\left(Y_{y}\right)+\lambda_{2}\delta_{2}\left(Y_{s}\right)\\ \delta_{1}\left(Y_{y}\right)=\frac{1-e^{-0.1Y_{y}}}{1+e^{-0.1Y_{y}}}\in \left(0,1\right)\\ \delta_{2}\left(Y_{s}\right)=\frac{Y_{s}}{N}\in \left[0,1\right] \end{array}\right.$$

$\lambda_{1},\lambda_{2}\in \left(0,1\right)$. The weighting coefficients $\lambda_{1}$ and $\lambda_{2}$ are user-configurable parameters that represent the decision-making preferences of traffic managers, rather than model parameters subject to optimization. $\lambda_{1}$ is primarily concerned with enhancing overall system efficiency by minimizing the average delay per person, while $\lambda_{2}$ prioritizes public transportation by reducing the number of stops. Traffic managers have the flexibility to dynamically adjust the ratio of these two coefficients in response to actual situational requirements, such as alleviating congestion during peak hours and improving the punctuality of public transport services.

### 5.4. Fault-Tolerance Mechanism

To address potential equipment failures, a three-tiered contingency protocol is implemented.

- Normal Operation: Fuse GPS positioning with multi-modal detector data (e.g., video/loop counts). Cross-validation minimizes measurement errors [27].
- Partial Failure: If one detector type fails (e.g., video sensor), retain functional sources (e.g., GPS + loops) and trigger an alert.
- Complete Failure: If all detectors malfunction,
  (a) Activate high-priority alarm;
  (b) Revert to the social vehicle arterial coordination scheme (baseline strategy);
  (c) Maintain current signal timing until recovery.

## 6. Deep Reinforcement Learning Model

This section further introduces the model in this chapter in two parts: virtual environment construction and deep reinforcement learning model training and solving. The virtual environment construction part is primarily focused on utilizing real road network environments and traffic data to establish a virtual simulation platform through Vissim. The model training and solving part mainly outlines the solution process.

### 6.1. Simulation Environment Construction

Constructing a traffic simulation model is essential for obtaining the subsequent state and reward value after taking an action. This paper utilizes dynamic acquisition of action evaluation parameters through simulation. A real scenario on Huancheng South Road in Kunming, Yunnan was selected to construct the Vissim simulation environment. The section spans approximately 1.3 km and includes four intersections. Signal control is divided into four main phases: morning peak (7:00–10:00), daytime (10:00–16:30), evening peak (16:30–20:30), and nighttime (20:30–7:00). Each phase has two signal phases with specific control schemes, as detailed in Table 2.

**Figure 7:**
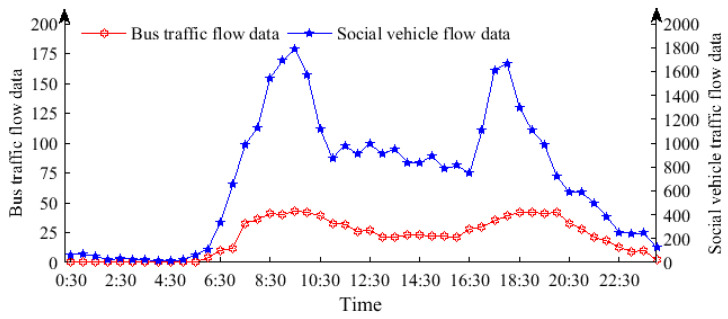
Traffic flow analysis.

In this study, α is set to 0.05, and the variation of delay is considered equally important as the variation of stop times at intersections; the setting of $\lambda_{1}=\lambda_{2}=0.5$ in Formula (10) is grounded in the principle of “efficiency and fairness in equal measure” within the domain of traffic control [28]. This reflects a balance between the passage efficiency for social vehicles and the prioritization of public transportation. In practical applications, it is essential for managers to adjust this ratio according to specific optimization objectives. There are a total of four intersections denoted by N=4. The maximum green light time $g_{\max}^{i}$ for each intersection is as follows: 75 s, 95 s, 75 s, and 75 s; the minimum green light time $g_{\max}^{i}$ for all four intersections is set at 28 s. The acquisition of $t_{a}^{i}$ primarily relies on car-mounted GPS devices. Buses operate within the test area from 6:30 to 23:30. Dynamic traffic information for the test section was collected through video acquisition and manual research, as depicted in Figure 7.

After analyzing the operational characteristics of bus services, the mainline coordinated phase difference was obtained and is presented in Table 3 (where only data within the operating hours of buses were selected for nighttime). It is evident that due to the impact of bus dwell time, there exists a significant disparity between the phase difference of buses and that of other vehicles on the mainline.

**Figure 8:**
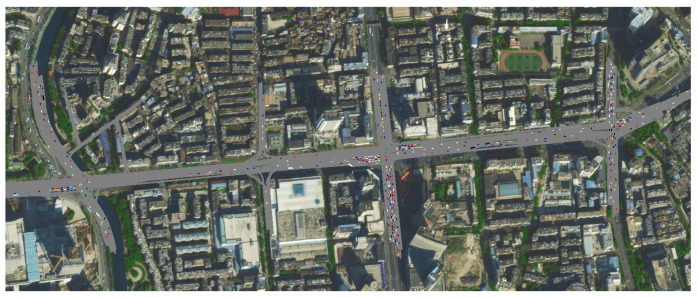
Traffic simulation model.

A traffic simulation model was developed based on road traffic organization. Through the secondary development of Vissim software (PTV Vissim 2020), real-time interaction between deep reinforcement learning decision-making and the simulation model was achieved. The modeling effect is illustrated in Figure 8.

### 6.2. Deep Reinforcement Learning Model

Deep reinforcement learning can be mainly divided into three categories: value-based, policy-gradient based, and search-based supervised. Value-based methods are simple but cannot directly generate action decisions and are limited to discrete action spaces. Search-based methods require additional human intervention to facilitate decision-making and lack theoretical foundations. Policy-gradient-based methods parameterize the policy and select the best expected policy to achieve direct decision-making from state to action. Considering value-based deep reinforcement learning, such as Deep Q Networks (DQNs), it cannot represent the execution of random policies based on probabilities and requires a discrete action space, making it difficult to apply in high-dimensional continuous state decision-making scenarios. Policy-gradient is a measure that allows neural networks to directly output probability decisions, enabling different execution strategies for different states. Deterministic policy gradient ensures that actions are determined and unique in certain states, which can further reduce sample requirements and improve computational efficiency. Therefore, based on the practical needs of traffic control, and in combination with the previous method introduction, DDPG is used as the solving algorithm (as presented in Algorithm 1).

**Figure 9:**
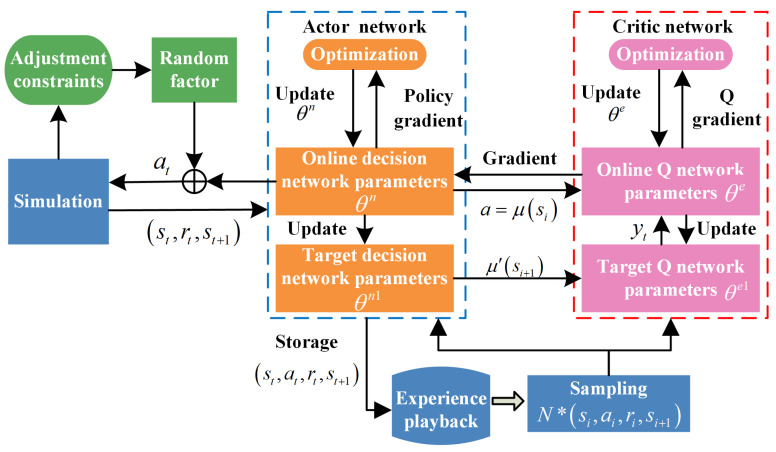
DDPG algorithm flowchart.

As shown in Figure 9, DDPG consists of an Actor decision network and a Critic evaluation network. The Actor decision network includes both online and target decision networks, which use deterministic decisions to obtain the next action from the current state and randomly select values within its range as the action amount, with the random factor set to quickly find the optimal value within the action value range. The Critic evaluation network mainly consists of online and target Q networks to overcome overfitting problems in the optimization process of Q-learning. The two networks are updated alternately using Equation (10) to measure the quality of the action taken.
**Algorithm 1** Deep deterministic policy gradient algorithm.**Input I: Status and reward $\left(s_{t},r_{t},s_{t+1}\right)$****Output I: Action $a_{t}\to \Delta t_{i}$**1. for each bus, from t=1 to t=T;2. Initial test system parameters, including network parameters, reward and punishment functions, etc.3. Combine with adjustment constraints and random factor, this model determined the action $a_{t}$, $a_{t}\in A$, and transfer the action into the simulation part.4. The simulation part executes action $a_{t}$, and it will receive the reward value $r_{t}$ and the new status $s_{t+1}$.5. If the sample pool overflows, then delete the earliest sample records in chronological order.6. The actor network will put the $\left(s_{t},a_{t},r_{t},s_{t+1}\right)$ into experience playback, which supplies the train data for the online network.7. Sampling from the experience pool, gain *N* sets of sample data $\left(s_{t},a_{t},r_{t},s_{t+1}\right)$ as the training set for the online actor network and Q network.8. Use the standard BP method to calculate the gradient of the online Q network.9. Update the parameter $\theta^{e}$ of the online Q network.10. Calculate the policy gradient (PG) of the actor network.11. Update the parameter $\theta^{n}$ of the online actor network.12. Update the parameters n1,e1 of the target network.13. End for.

## 7. Experiment Analysis

### 7.1. Hyperparameter Configuration

Taking into consideration the need to strike a balance between learning rate and retention of previous training effects, as well as the significance of balancing experience and rewards, appropriate network structures and parameters were selected through multiple experiments in this algorithm. The comprehensive parameter settings for the DDPG-based arterial coordination control are categorized and detailed in Table 4.

### 7.2. State Space Analysis and Training Optimization

In the modeling and algorithmic training process for traffic signal optimization, the rational design of state space dimensionality and the enhancement of training stability and efficiency are critical components. This section first analyzes the number of intersections and their corresponding state space dimensionality, followed by the introduction of dimensionality reduction methods to ensure algorithmic scalability across different scale scenarios. Subsequently, an adaptive exploration strategy and prioritized experience replay mechanism based on temporal difference error are proposed to accelerate training convergence and improve policy quality. Finally, multiple technical measures, including gradient clipping, soft updates, and convergence criteria, are detailed to ensure robust model training and effective convergence.

Dimensional Constraints Analysis: Based on arterial coordination standards [29], effective signal coordination requires

(11) $$300\;m\leq d_{i}\leq 800\;m\;(\mathrm{intersection}\;\mathrm{spacing})$$

For a 1.5 km corridor (L=1500m), the maximum number of intersections is

(12) $$n_{\max}=\left\lfloor \frac{L}{d_{\min}}\right\rfloor +1=\left\lfloor \frac{1500}{300}\right\rfloor +1=6$$

In practical applications, n∈[3,5] covers 92% of urban arterials. Our Kunming case study (L=1300m,n=4) falls within this optimal range, where the state space $S=(s_{1},\ldots ,s_{n})$ maintains computational efficiency without requiring dimensionality reduction.

State Space Compression: When the number of intersections exceeds the standard coordination limits (n≥7), Principal Component Analysis (PCA) is implemented to prevent dimensionality explosion.

(13) $$\frac{\sum_{i=1}^{k}\lambda_{i}}{\sum_{i=1}^{d}\lambda_{i}}\geq 0.95$$

where $\lambda_{i}$ denotes the eigenvalues of the covariance matrix. This preserves 95% of the variance while effectively compressing the state space dimensionality.

Adaptive Exploration Strategy: Ornstein–Uhlenbeck noise with time-decaying amplitude is applied during action sampling:

(14) $$\sigma_{t}=0.02+\left(1.8-0.02\right)\cdot e^{-0.0001t}=0.02+1.78e^{-0.0001t}$$

This configuration promotes extensive exploration (σ>1.5) during initial training while converging to minimal disturbance (σ<0.1) in later stages.

Prioritized Experience Replay: Transition sampling prioritization is weighted by temporal difference error magnitude.

(15) $$P\left(i\right)=\frac{\left(\right|\delta_{i}{\left|+10^{-5}\right)}^{0.6}}{\sum_{j}\left(\right|\delta_{j}{\left|+10^{-5}\right)}^{0.6}}$$

Training Stability Measures: To ensure robust convergence, multiple stabilization techniques are implemented.

- Gradient Clipping: Constrain policy gradients to ∥∇Q∥<5.0 to prevent explosion.
- Soft Updates: Target network parameters updated as $\theta_{\mathrm{target}}\leftarrow 0.01\theta_{\mathrm{online}}+0.99\theta_{\mathrm{target}}$.
- Convergence Criterion: Training terminates when $\frac{|Q_{t}-Q_{t-1}|}{Q_{t-1}}<0.05$ for 20 consecutive iterations.

In summary, this approach integrates real-world traffic constraints with reinforcement learning characteristics to develop a scientifically grounded state space management and training optimization framework, ensuring effective algorithmic performance in complex traffic scenarios while maintaining training efficiency.

### 7.3. Deep Reinforcement Learning Model Training

**Figure 10:**
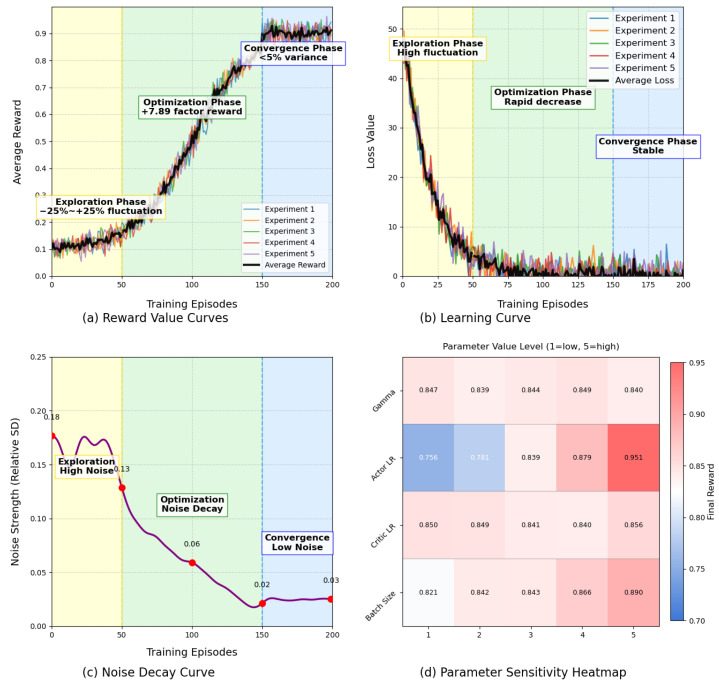
DDPG train results.

Figure 10 systematically examines the key aspects of the reinforcement learning model training process through four integrated charts. The chart in the upper left corner (a) illustrates the reward value curve, tracking five distinct experimental runs along with their average values over training rounds. This chart delineates three distinct phases: during the exploration phase (rounds 0–50, yellow background), as the model investigates its environment, the reward value exhibits significant fluctuations (±25%); subsequently, in the optimization phase (rounds 50–150, green background), as strategies improve, there is a remarkable increase in reward value by a factor of 7.89; finally, in the convergence phase (rounds 150–200, blue background), the reward value stabilizes with minimal variance (<5%), indicating that learning has reached a mature stage.

Complementing this perspective on rewards, the chart in the upper right corner (b) presents the learning curve that reflects reductions in model error. During the high-variability exploration phase (yellow), loss values fluctuate considerably; however, this is followed by a rapid decrease in error during the optimization phase (green). Ultimately, this process stabilizes at a very low loss value during the convergence phase (blue), with all five experiments achieving loss values below 10% by episode 200.

The noise analysis depicted in the lower left corner (c) quantifies how environmental complexity gradually diminishes over time. The noise intensity—measured as relative standard deviation—decreases from an initial level of 0.13 during chaotic exploration to between 0.02 and 0.06 throughout convergence periods. This observed attenuation of noise facilitates model adaptation and is particularly evident from episodes 50 to 150 when intentional reduction of noise contributes significantly to achieving optimal rewards as illustrated in chart (a).

The heatmap located in the lower right corner (d) assesses the sensitivity of hyperparameters based on the final reward value, with the horizontal and vertical axes representing varying levels of these parameters (1 indicating low and 5 indicating high). Two prominent patterns emerge: first, the actor learning rate (upper right corner) exhibits a significant impact, as its level 5 setting elevates the reward value to 0.951; second, the batch size (lower right corner) reveals a concave relationship, with performance peaking at a medium size. This structured sensitivity analysis effectively quantifies the degree of influence that each parameter exerts, which is crucial for optimizing performance tuning.

### 7.4. Verification of Arterial Signal Switching Strategy

**Figure 11:**
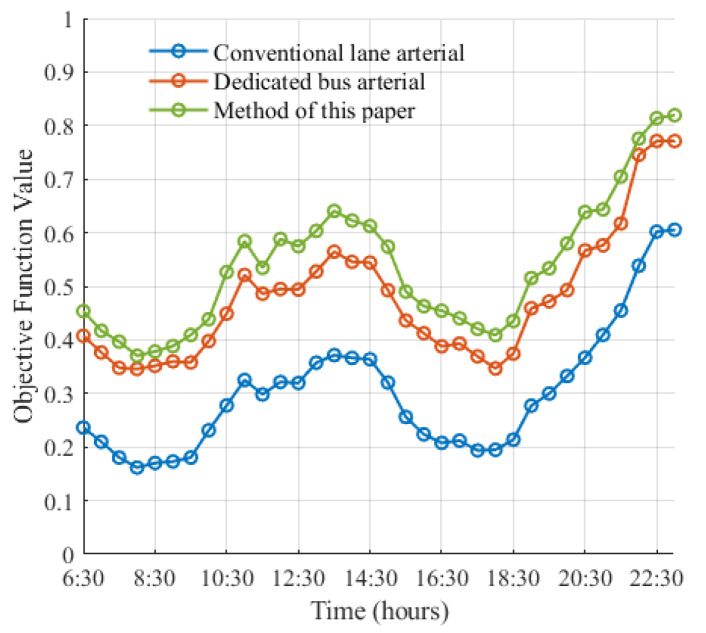
Optimization target function value.

This study conducted comparative experiments on three groups of coordinated control for arterial roads: Conventional lane arterial coordination, dedicated bus arterial coordination, and the arterial coordination proposed in this chapter. Realistic traffic simulations were utilized for case analysis. After conducting ten simulations and averaging the results, it was determined that the target values controlled by the arterial coordination proposed in this chapter were found to be optimal at all stages (as depicted in Figure 11), with optimization improvements of 29.77% and 8.11% compared to the other two arterial coordination methods, respectively.

**Figure 12:**
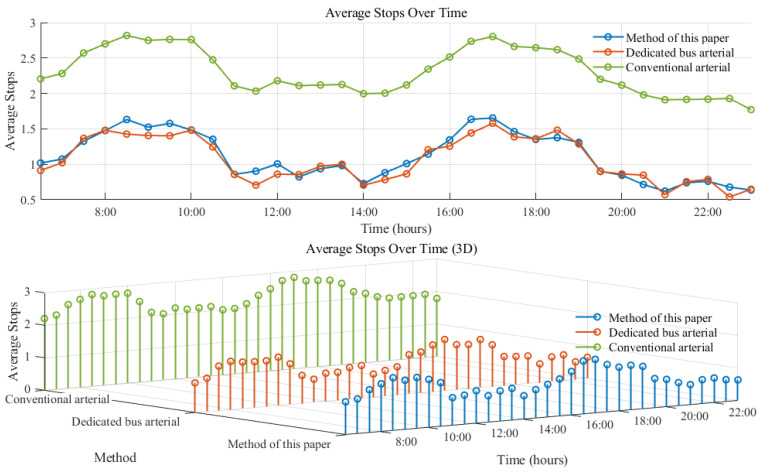
Average stops over time.

**Figure 13:**
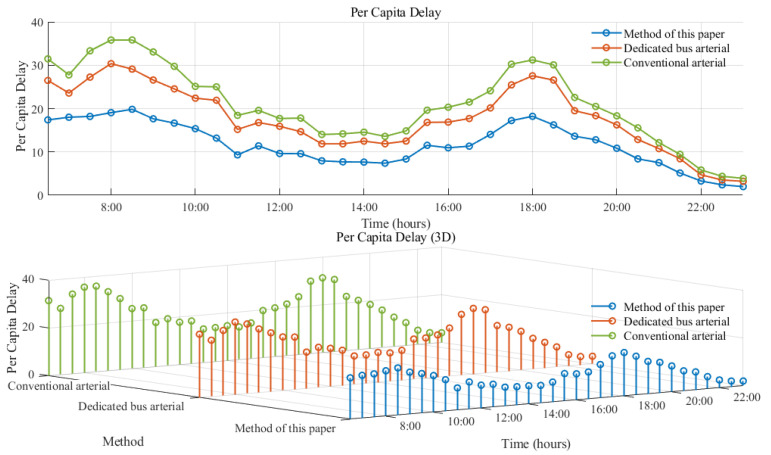
Per capita delay.

As depicted in Figure 12 and Figure 13, the average number of stops at the bus intersection under social vehicle arterial coordination is 2.30. In contrast, under dedicated bus lane arterial coordination and the arterial coordination discussed in this chapter, the average number of stops are 1.06 and 1.10, respectively. Therefore, it can be inferred that the arterial coordination control method proposed in this paper can better ensure the efficiency of bus operation. Simultaneously, an analysis of road delay per capita under the three states reveals that the average delay for dedicated bus lane arterial coordination is 17.63 s per day, while for social vehicle arterial coordination it is 14.91 s per day, and for the arterial coordination presented in this chapter it is 10.82 s per day. Compared to the previous two methods, our proposed arterial coordination method shows improvements of 38.63% and 27.43%, respectively, indicating its ability to enhance bus traffic efficiency while reducing negative impacts on social vehicles.

This paper presents a bus arterial green-wave control method based on deep reinforcement learning. The method alternates between social vehicle arterial coordination and dedicated bus lane arterial coordination. Compared to single social vehicle arterial coordination or dedicated bus lane arterial coordination, the proposed method improves the average delay per capita by 38.63% and 27.43%, respectively. Simulation tests based on actual scenarios demonstrate that the proposed arterial green-wave control method, which coordinates the demands of social vehicle arterial and dedicated bus lane arterial in an alternate manner, surpasses the limitations of a single green-wave coordination scheme. Additionally, by integrating with a deep reinforcement learning framework suitable for high-dimensional continuous traffic states, the proposed method achieves real-time decision-making under continuous state and action values.

### 7.5. Saturation Scenario Applicability Analysis

To validate the robustness of the proposed method under varying traffic saturation levels, this section defines saturation states based on the GB 50647-2011 Urban Road Intersection Planning Specifications: critical saturation (0.90–1.0); moderate saturation (1.0–1.2); high saturation (1.20–1.35). High saturation risks queue overflow and intersection gridlock, potentially causing control strategies to fail. Thus, the moderate saturation level (saturation ratio = 1.1) is selected for comparative analysis.

The experimental setup is detailed in Table 5, leveraging data and models from existing sections.

**Figure 14:**
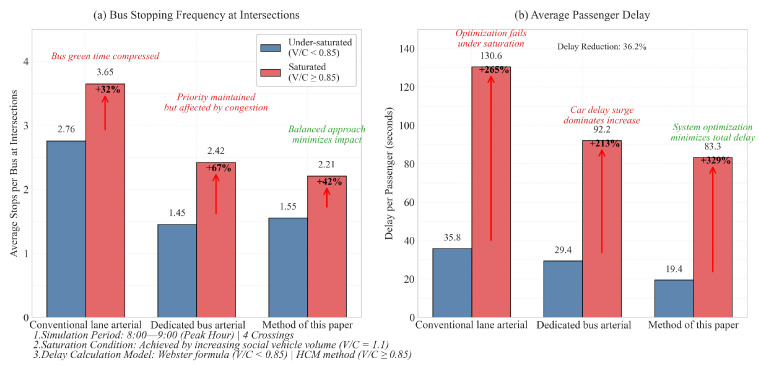
Saturation state comparison.

The experimental results (shown in Figure 14) demonstrate significant performance differences across control strategies under varying saturation conditions. For bus stopping frequency at intersections, the Conventional arterial strategy exhibited the highest stops in both under-saturated (2.76 stops/bus) and saturated conditions (3.65 stops/bus), while the Dedicated bus arterial strategy showed moderate performance (1.45 stops/bus under-saturated, 2.42 saturated). The proposed method achieved the best performance with 1.55 stops/bus under-saturated and 2.21 stops/bus in saturated state. Regarding passenger delay, the conventional strategy incurred the longest delays (35.82 s under-saturated, 130.60 s saturated), with the dedicated bus strategy showing improved efficiency (29.42 s, 92.18 s). The proposed method maintained the lowest delays across both operational states (19.44 s under-saturated, 83.34 s saturated), demonstrating its effectiveness in balancing bus priority and system-wide traffic flow optimization.

## 8. Conclusions

This paper presents a green-wave control method for bus trunk lines based on deep reinforcement learning, which alternates between coordinating the needs of social vehicles and those of bus lanes. Compared to single coordination strategies focused solely on either social vehicles or bus lanes, the proposed method achieves a reduction in average delay per person by 38.63% and 27.43%, respectively. Further analysis under saturation scenarios indicates that this approach reduces the average delay per person by 29.7% (83.34 s vs. 130.60 s) when compared to coordination for social vehicles and by 9.6% (83.34 s vs. 92.18 s) relative to dedicated bus lane coordination; it also decreases the number of bus stops by 39.5% (2.21 times vs. 3.65 times) and by 8.7% (2.21 times vs. 2.42 times), respectively. Simulation tests conducted in real-world scenarios demonstrate that the proposed green-wave control method effectively addresses the limitations inherent in traditional single green-wave coordination schemes through its alternating focus on both social vehicle demands and bus lane requirements. Moreover, by integrating a deep reinforcement learning framework tailored for high-dimensional continuous traffic states, this method facilitates real-time decision-making within continuous state and action value environments.

## Figures and Tables

**Table 1:** K-S test of travel time distribution.

		V1	V2
Cases		400	400
Normal parameter a,b	Average value	0.183841272	0.107313444
	Standard deviation	0.142599558	0.117999785
Most extreme difference	Absolute	0.132	0.202
	Positive	0.132	0.202
	Negative	−0.107	−0.182
Test statistics		0.132	0.202
Asymptotically significant (two-tailed)		0.076	0.101

**Table 2:** Intersection signal timetable (unit/s) distribution.

NO.	Yellow	All Red	Morning Peak and Evening Peak			Daytime			Nighttime		
			Cycle	Green	Red	Cycle	Green	Red	Cycle	Green	Red
1	3	2	117	55	58	97	46	47	77	36	37
2	3	2	117	83	30	97	58	35	77	45	28
3	3	2	117	63	50	97	50	43	77	38	35
4	3	2	117	66	47	97	52	41	77	40	33

**Table 3:** Phase difference comparison (unit/s) distribution.

Phases	Type	Intersection 1	Intersection 2	Intersection 3	Intersection 4
Morning Peak and Evening Peak	Social vehicle	0	43	10	53
	Dedicated bus	0	58	14	69
Daytime	Social vehicle	0	55	94	58
	Dedicated bus	0	68	2	72
Nighttime	Social vehicle	0	36	35	35
	Dedicated bus	0	48	40	49

**Table 4:** DDPG hyperparameter configuration.

Category	Symbol	Value	Description
Optimization	γ	0.9	Discount factor for future rewards
	$\alpha_{\mathrm{actor}}$	$10^{-4}$	Actor network learning rate
	$\alpha_{\mathrm{critic}}$	$10^{-5}$	Critic network learning rate
	*T*	200	Total training iterations
	*B*	32	Batch size
Structural	$N_{\mathrm{hidden}}$	3×512	Hidden layer configuration
	M	$10^{3}$	Experience replay buffer size
	$\eta_{\mathrm{PCA}}$	0.95	PCA variance threshold
Stabilization	$\sigma_{\max}$	1.80	Initial OU noise variance
	$\sigma_{\min}$	0.02	Minimum OU noise variance
	κ	$10^{-4}$	Noise decay coefficient
	${∥\nabla Q∥}_{\max}$	5.0	Gradient clipping threshold
	τ	0.01	Target network update coefficient
	$\Delta Q_{\mathrm{th}}$	5%	Q-value fluctuation limit
	$N_{\mathrm{conv}}$	20	Stable iterations required

**Table 5:** Experimental setup parameters.

Parameter Category	Comparison Group	Experimental Group
Time Period	8:00–9:00	Same as comparison group
Social Vehicle Flow	1987.98 veh/h	3219.33 veh/h
Bus Flow	Same as Section 6.1	Same as comparison group
Road Configuration	Same as Section 6.1	Same as comparison group
Actual Saturation Flow Rate	1463.33 veh/h/lane	Same as comparison group
Calculated Saturation Ratio	0.68 (slightly congested)	1.1 (moderate saturation)

## Data Availability

The data that support the findings of this study are available from the corresponding author [Fenghua Zhu] upon reasonable request.

## Abbreviations

The following abbreviations are used in this manuscript:

DDPG	Deep Deterministic Policy Gradient
MDP	Markov Decision Process
GPS	Global Positioning System

## References

[1] Guler S.I., Menendez M. (2014). Analytical formulation and empirical evaluation of pre-signals for bus priority. Transp. Res. Part B Methodol..

[2] Truong L.T., Currie G., Wallace M., De Gruyter C., An K. (2019). Coordinated Transit Signal Priority Model Considering Stochastic Bus Arrival Time. IEEE Trans. Intell. Transp. Syst..

[3] Colombaroni C., Fusco G., Isaenko N. (2020). A Simulation-Optimization Method for Signal Synchronization with Bus Priority and Driver Speed Advisory to Connected Vehicles. Transp. Res. Procedia.

[4] Zhang X., He Z., Zhu Y., You L. (2023). DRL-based adaptive signal control for bus priority service under connected vehicle environment. Transp. B Transp. Dyn..

[5] Li G., Li S., Li S., Qin Y., Cao D., Qu X., Cheng B. (2020). Deep reinforcement learning enabled decision-making for autonomous driving at intersections. Automot. Innov..

[6] Yoon J., Ahn K., Park J., Yeo H. (2021). Transferable traffic signal control: Reinforcement learning with graph centric state representation. Transp. Res. Part C Emerg. Technol..

[7] Liang X., Du X., Wang G., Han Z. (2019). A deep reinforcement learning network for traffic light cycle control. IEEE Trans. Veh. Technol..

[8] Wang Y., Guo Y. (2019). Signal Priority Control for Trams Using Deep Reinforcement Learning. Acta Autom. Sin..

[9] Shi W., Yu C., Ma W., Wang L., Nie L. (2020). Simultaneous optimization of passive transit priority signals and lane allocation. KSCE J. Civ. Eng..

[10] Bie Y., Liu Z., Wang H. (2020). Integrating Bus Priority and Presignal Method at Signalized Intersection: Algorithm Development and Evaluation. J. Transp. Eng. Part A Syst..

[11] Zeng X., Zhang Y., Jiao J., Yin K. (2020). Route-based transit signal priority using connected vehicle technology to promote bus schedule adherence. IEEE Trans. Intell. Transp. Syst..

[12] Li J., Liu Y., Zheng N., Tang L., Yi H. (2021). Regional coordinated bus priority signal control considering pedestrian and vehicle delays at urban intersections. IEEE Trans. Intell. Transp. Syst..

[13] Long M., Zou X., Zhou Y., Chung E. (2022). Deep reinforcement learning for transit signal priority in a connected environment. Transp. Res. Part C Emerg. Technol..

[14] Li H., Li S., Zhang X., Tong P., Guo Y. (2023). Dynamic signal priority of the self-driving bus at an isolated intersection considering private vehicles. Sci. Rep..

[15] Xu M., Zhai X., Sun Z., Zhou X., Chen Y. (2023). Multiagent control approach with multiple traffic signal priority and coordination. Transp. Eng. Part A Syst..

[16] Hu X., Chen X., Guo J., Dai G., Zhao J., Long B., Zhang T., Chen S. (2023). Optimization model for bus priority control considering carbon emissions under non-bus lane conditions. J. Clean. Prod..

[17] Dai G., Wang H., Wang W. (2015). A bandwidth approach to arterial signal optimisation with bus priority. Transp. A Transp. Sci..

[18] Xu M., An K., Ye Z., Wang Y., Feng J., Zhao J. (2018). A bi-level model to resolve conflicting transit priority requests at urban arterials. IEEE Trans. Intell. Transp. Syst..

[19] Wu K., Lu M., Guler S.I. (2020). Modeling and optimizing bus transit priority along an arterial: A moving bottleneck approach. Transp. Res. Part C Emerg. Technol..

[20] Chen Y.H., Cheng Y., Chang G.L. (2023). Incorporating bus delay minimization in design of signal progression for arterials accommodating heavy mixed-traffic flows. J. Intell. Transp. Syst..

[21] Ou S., An K., Ma W., Hegyi A., Van Arem B. (2024). Stochastic-priority-integrated signal coordination considering connected bus operation uncertainties. Transp. B Transp. Dyn..

[22] Shao Y., Sun J., Kan Y., Tian Y. (2024). Operation of dedicated lanes with intermittent priority on highways: Conceptual development and simulation validation. J. Intell. Transp. Syst..

[23] Seman L.O., Koehler L.A., Camponogara E., Kraus W. (2020). Integrated headway and bus priority control in transit corridors with bidirectional lane segments. Transp. Res. Part C Emerg. Technol..

[24] Anderson P., Daganzo C.F. (2020). Effect of transit signal priority on bus service reliability. Transp. Res. Part B Methodol..

[25] Pallela S.S., Mehar A. (2024). Analysis of Time Headway Characteristics at the Curbside Bus Stop on Multi-Lane Divided Urban Arterials under Mixed Traffic Conditions. KSCE J. Civ. Eng..

[26] Thodi B.T., Chilukuri B.R., Vanajakshi L. (2022). An analytical approach to real-time bus signal priority system for isolated intersections. J. Intell. Transp. Syst..

[27] (2023). Urban Road Traffic Operation Data Fusion Specification. Chongqing Local Standard.

[28] (2018). Road Traffic Signal Control Modes—Part 6: Control Rules for Priority Passage of Buses at Intersections. Public Security Industry Standard of China.

[29] (2011). Code for Planning of Urban Road Intersections. National Standard of China.

